# Evaluation and management of primary sclerosing cholangitis patients awaiting liver transplantation

**DOI:** 10.1016/j.jhepr.2026.101748

**Published:** 2026-01-30

**Authors:** Alexandra D. Frolkis, Jeremy S. Nayagam, Alessandro Parente, Matthew Seager, Matthew D. Sadler, Aldo J. Montano-Loza, Deepak Joshi

**Affiliations:** 1 Division of Gastroenterology and Hepatology, Department of Medicine, Cumming School of Medicine, University of Calgary, Calgary, AB, Canada; 2 Institute of Liver Studies, King’s College Hospital NHS Foundation Trust London, London, United Kingdom; 3 Faculty of Life Sciences and Medicine, School of Immunology and Microbial Sciences, The Roger Williams Institute of Liver Studies, King's College London, London, United Kingdom; 4 Department of Radiology, Princess Alexandra Hospital, Brisbane, Australia; 5 Division of Gastroenterology and Liver Unit, Department of Medicine, University of Alberta, Edmonton, Alberta, Canada

**Keywords:** Primary sclerosing cholangitis, liver transplantation, management

## Abstract

Primary sclerosing cholangitis (PSC) is a rare, progressive cholestatic liver disease characterised by biliary strictures, increased risk of cholangiocarcinoma, and a strong association with inflammatory bowel disease. Despite its low prevalence, PSC accounts for up to 17% of all liver transplants (LT) performed globally. Transplant-free survival ranges from a median of 9 to 21 years from diagnosis. LT is the only definitive treatment, with post-LT survival in PSC exceeding 80% at 5 years. However, PSC recurs in approximately 25% of individuals. The absence of disease-modifying therapy necessitates timely LT in select cases of hepatic decompensation, refractory pruritus, recurrent cholangitis, or selected early-stage malignancies. However, PSC poses unique challenges in pre-transplant assessment and timing due to its clinical heterogeneity and the suboptimal performance of conventional prognostic tools, such as the MELD score. Although PSC-specific scores offer improved risk stratification, they are not integrated into routine LT algorithms. This review synthesises the current landscape of prognostic modelling in PSC, focusing on validated tools and their role in transplant decision making. We examine the range of LT indications in PSC and explore the interplay between PSC and inflammatory bowel disease, with particular focus on malignancy surveillance and immunosuppression planning. We review the transplant evaluation process, including hepatobiliary imaging, endoscopy, and multidisciplinary approaches to high-grade stricture evaluation and oncologic risk assessment. By integrating contemporary evidence and expert opinion, this review aims to guide clinicians in the nuanced assessment and management of patients with PSC in the peri-transplant period, emphasising the need for individualised risk assessment and early referral to transplantation centres with hepatobiliary surgery and transplant expertise.

## Graphical abstract

**Figure:**
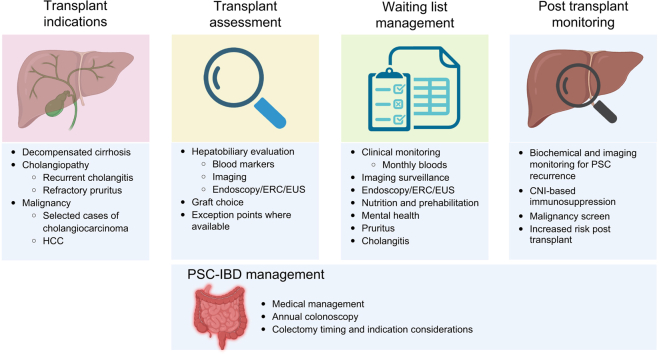

## Introduction

Primary sclerosing cholangitis (PSC) is a chronic, progressive cholestatic liver disease characterised by multifocal biliary strictures with increased risk of cholangitis, fibrosis, cirrhosis with portal hypertensive complications, and hepatobiliary malignancy. The incidence and prevalence of PSC are increasing.^1^ The worldwide pooled prevalence is estimated between <1 and 31.7 per 100,000, and PSC is more common in European and North American countries.^2^ PSC shows a male predominance of 65-75%^3^ and has a strong association with inflammatory bowel disease (IBD). Though it can occur at any age, median and mean age at diagnosis range from 33 to 45 years.[3], [4], [5] Transplant-free survival ranges from 9.7 to 20.6 years.^5^ Despite its rarity, it accounts for 5-17.3% of liver transplant (LT) procedures in European countries.[6], [7], [8] As there are no approved disease-modifying therapies, the only definitive treatment is LT. Survival after LT for PSC is favorable and exceeds 80% at 5 years.^9^ However, even with LT, PSC can recur (rPSC). The principal indications for LT in PSC include hepatic decompensation, recurrent cholangitis, and refractory pruritus unresponsive to medical therapy. In some centres, selected cases of high-grade biliary dysplasia and cholangiocarcinoma (CCA) are qualifying indications.^10^ Given the potential for rapid deterioration, timely referral for transplant evaluation is essential. The purpose of this review is to highlight transplant assessment and peri-transplant management of patients with PSC.

We conducted a literature search using OVID/EMBASE to identify relevant studies. The core search strategy combined (“primary sclerosing cholangitis” OR “PSC”) AND (“LT” OR “transplant∗”). Additional terms related to each subsection were additionally included. Titles and abstracts were screened for relevance to this review. Reference lists of included studies were examined to capture additional pertinent studies.

## Prognostic assessment for transplant timing

**Fig. 1:**
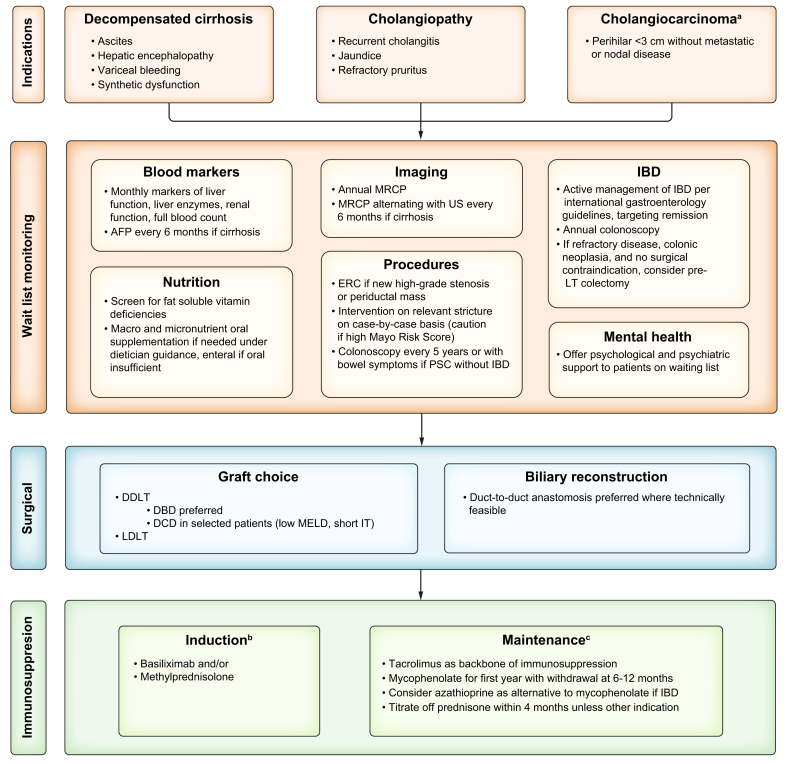
Flowchart for liver transplantation in PSC. AFP, alpha fetoprotein; DBD, deceased brain donor; DCD, donation after circulatory death; DDLT, deceased donor liver transplantation; ERC, endoscopic retrograde cholangiography; IBD, inflammatory bowel disease; IT, ischemic time; LDLT, living donor liver transplantation; LT, liver transplantation; MELD, model for end stage liver disease; MRCP, magnetic resonance cholangiopancreatography; PSC, primary sclerosing cholangitis; US, ultrasound. ^a^Centre-specific with strict inclusion criteria. ^b^Basiliximab two doses of 20 mg at postoperative day 0 and 4 and/or methylprednisolone 1 g at postoperative day 0 then 16 mg daily IV until tolerated orally at 20 mg with tapering of 5 mg every 2 weeks. Long-term steroid use without another indication is not recommended. ^c^Tacrolimus trough goal 7–10 ng/ml in first month; 4–8 ng/ml in months 1–12; 4 ng/ml after year 1; mycophenolate 1 g twice daily with withdrawal after 6–12 months unless used for renal-sparing immunosuppression; azathioprine 1–1.5 mg/kg/day.

Assessing prognosis and timing for LT assessment in PSC is uniquely challenging due to its heterogeneity, a markedly increased and unpredictable risk of biliary malignancies, multiple indications for LT (Fig. 1), and the limitations of conventional liver disease models to predict clinical outcomes. It is well established that model for end-stage liver disease (MELD) and its derivatives (including MELD-Na, MELD 3.0, and UKELD) may underestimate the burden of disease in PSC as the objective markers in these scores (universally based on international normalised ratio, bilirubin, and creatinine) do not capture unique complications of PSC such as cholangitis, pruritus, impact on quality of life, and risk of hepatobiliary malignancies.^11^ PSC requires an integrative approach encompassing biochemical markers, cholangiographic findings, and clinical symptoms, each of which provides complementary yet distinct insights into disease progression. Accurate prognostication is essential not only for identifying patients at risk of hepatic decompensation or cholangiocarcinoma but also for optimizing the timing of LT referral. While MELD-based systems guide allocation, they fail to capture the full burden of PSC, prompting the development of PSC-specific tools. Multiple PSC-specific prognostic scores exist; however, these scores identify patients at high risk of adverse outcomes and are not routinely integrated into transplant assessment frameworks^12^ (Table 1). However, they are associated with graft loss after LT.^12^

**Table 1:** Prognostic scores in primary sclerosing cholangitis.

Score	Key variables	Primary outcome predicted
**Biochemistry based**		
MELD/MELD-Na/MELD 3.0/UKELD	INR, bilirubin, creatinine MELD-Na and UKELD additionally include sodium MELD 3.0 additionally includes albumin	Waitlist mortality, decompensation risk
Revised Mayo	Age, bilirubin, albumin, AST, variceal bleeding	4-year mortality
Amsterdam Oxford Model	PSC subtype, age at diagnosis, albumin, platelets, AST, ALP, bilirubin	Transplant-free survival
UK-PSC (short-term/long-term)	Short-term: bilirubin, albumin, platelets, haemoglobin Long-term: age, bilirubin, ALP, albumin, platelets, variceal haemorrhage, extrahepatic disease	2-year and 10-year survival
PREsTo	Bilirubin, albumin, ALP ( × ULN), platelets, AST, haemoglobin, sodium, age, disease duration	Decompensation
ELF test	Hyaluronic acid, TIMP-1, PIIINP	Transplant-free survival

**Biochemistry with portal hypertensive complication**		
Revised Mayo	Age, bilirubin, albumin, AST, variceal bleeding	4-year mortality
UK-PSC (short-term/long-term)	Short-term: bilirubin, albumin, platelets, haemoglobin Long-term: age, bilirubin, ALP, albumin, platelets, variceal haemorrhage, extrahepatic disease	2-year and 10-year survival

**Cholangiography based**		
Anali MRI	MRI features: bile duct dilatation, liver dysmorphy, portal hypertension	Decompensation and transplant risk
DiStrict scores	Intra and extrahepatic duct dilatation	Transplant risk and mortality

ALP, alkaline phosphatase; AST, aspartate aminotransferase; ELF, enhanced liver fibrosis; INR, international normalisation ratio; MELD, model for end stage liver disease.

PIIINP, amino-terminal propeptide of type III procollagen; PREsTo, primary sclerosing cholangitis risk estimate tool; TIMP-1, tissue inhibitor of metalloproteinases-1; UKELD, United Kingdom model for end stage liver disease; UK-PSC, United Kingdom primary sclerosing cholangitis; ULN, upper limit of normal.

### Biochemistry-based models

The earliest prognostic tools (Table 1), including the original and revised Mayo PSC risk scores, were derived from tertiary centre cohorts and primarily aimed to predict short-term mortality.^13^^,^^14^ The revised Mayo model incorporates age, bilirubin, albumin, aspartate aminotransferase (AST), and history of variceal bleeding. While widely used, its utility is limited to forecasting 4-year mortality and does not predict decompensation or survival.^5^ A recent single-centre study of 109 adults with newly diagnosed primarily low-risk PSC (80% of cohort) found the Mayo risk score had excellent discrimination for LT or death.^15^ The earliest iteration involved histology, which is no longer necessary for the diagnosis of large duct disease and is not used routinely in clinical practice aside from in variant syndromes of PSC.^16^

To overcome these limitations, newer models have been developed. The Amsterdam-Oxford model (AOM)^17^ predicts transplant-free survival and is based on seven routinely available clinical and laboratory parameters: PSC subtype, age at diagnosis, albumin, platelets, alkaline phosphatase (ALP), AST, and bilirubin. Although validated in population-based cohorts, its predictive accuracy at diagnosis remains moderate (c-statistic 0.68). The AOM performance improves with yearly serial measurements with an acceptable predictive accuracy at 5 years from diagnosis of PSC (c-statistic 0.75).^18^ One quarter (25.4%) of patients shifted from a low-risk category (AOM less than 2.0) at diagnosis to a higher risk category (AOM greater than 2.0) after 5 years of follow-up, which was associated with a significant likelihood of needing LT or dying (hazard ratio [HR] 4.09; 95% CI 2.99-5.61).^18^ AOM can therefore be used to identify patients at higher risk of needing LT and can be repeated while a patient is waiting for LT as a means of risk stratification. The Mayo risk score overestimated the risk of death or LT when compared to the AOM in a cohort of patients from three tertiary care centres.^18^ The overestimation with the Mayo risk score was most pronounced in a cohort with AOM greater than 2 (high risk of death or need for LT). Therefore, compared to the AOM, the Mayo risk score is not as useful as a guide to determine if an individual patient should proceed with LT. The main challenge with the clinical use of AOM for timing LT, however, is that a significant proportion of patients do not require LT within this timeline; transplant-free survival at 1 year and 5 years after PSC diagnosis was 98.3% and 84.4%, respectively.^18^ This further supports its use serially throughout the disease course.

The UK-PSC risk scores, developed from a large multicentre cohort, provide both short-term (2-year) and long-term (10-year) prognostic estimates of death or need for LT after diagnosis.^19^ The short-term model includes bilirubin, albumin, platelets, and haemoglobin at diagnosis. The long-term model includes age at diagnosis; bilirubin, ALP, albumin, and platelets at 2 years after diagnosis; presence of extrahepatic disease at diagnosis; and variceal haemorrhage by 2 years after diagnosis. The long-term score shows robust discrimination (c-statistic 0.85), outperforming earlier models. The short-term and long-term scores showed an improved predictive accuracy for predicting death or transplantation compared to the revised Mayo and MELD scores.^19^ In both the short-term and long-term scores, bilirubin >50 umol/L had the highest HR (5.02, 95% CI 2.76-9.13 and 3.96, 95% CI 2.37-6.62, respectively) for death or LT.

Bilirubin is a variable used in all the biochemistry-based models and serves as a helpful marker to identify patients who should be considered for transplantation. An important caveat is that bilirubin is a marker of advanced disease, can fluctuate during the natural history of PSC, and can improve with antibiotic treatment and endoscopic biliary intervention. Therefore, the trajectory of bilirubin is a vital consideration.

The ELF (enhanced liver fibrosis) test is a non-invasive serum biomarker panel that predicts transplant-free survival in patients with PSC. It combines three direct markers of extracellular matrix turnover to reflect hepatic fibrogenesis: hyaluronic acid, tissue inhibitor of metalloproteinases-1, and the amino-terminal propeptide of type III procollagen. In a large, multicentre validation study, higher ELF scores were independently associated with an increased risk of LT or death, with a cut-off of ≥9.8 identifying high-risk patients with strong discriminative power (AUC 0.80).^20^

A recent advance in risk prediction is the PREsTo (primary sclerosing cholangitis risk estimate tool), a machine learning-based model derived using gradient boosting.^21^ PREsTo predicts hepatic decompensation and uses nine variables: bilirubin, albumin, ALP (x the upper limit of normal), platelets, AST, haemoglobin, sodium, age, and disease duration. It demonstrated excellent discrimination (c-statistic 0.90), outperforming MELD and Mayo scores. However, we are still awaiting data on its performance in predicting transplant-free survival.

### Cholangiographic-based models

Cholangiographic scores such as DiStrict and Anali use magnetic resonance cholangiopancreatography (MRCP) findings to quantify biliary damage and fibrosis. The DiStrict score, based on MRCP findings, quantifies intrahepatic and extrahepatic duct changes by scoring the presence and extent of strictures and upstream dilatation. Patients are classified from 0 to 8, with higher scores reflecting more severe ductal abnormalities.^22^ A DiStrict score ≥5 significantly predicts an increased risk of LT or liver-related death, with patients scoring 5–8 experiencing an 8.2-fold greater risk compared to those with lower scores.^22^

Similarly, the Anali score, derived from MRI, assesses intrahepatic bile duct dilatation, hepatic dysmorphy, and evidence of portal hypertension.^23^^,^^24^ Each feature is assigned a weighted score, and higher Anali scores are independently associated with increased rates of cirrhosis decompensation, LT, and mortality. An Anali score >2 combined with elevated liver stiffness measurement (LSM) by vibration-controlled transient elastography (VCTE) identifies patients at particularly high risk, with a 5-year cumulative adverse outcome rate of up to 38%.^23^ Both scoring systems emphasise structural deterioration of the biliary tree as a surrogate for progressive liver disease, offering a non-invasive means of predicting clinical endpoints. These risk scores can be used to identify at-risk patients in whom transplant referral should be considered.

Recently, the prospective validation of LSM by VCTE in PSC was presented in the FICUS study. Adult patients with PSC from 13 institutions were prospectively recorded at baseline and then annually for 5 years. Baseline LSM was strongly and independently (adjusted for age, sex, bilirubin, and ALP) linked to the risk of death or LT (adjusted HR 1.05 per kPa increase).^25^ Observed LT-free survival at 5 years was 93.8% in PSC with LSM 2.5–9.5 kPa, 79.0% with LSM 9.6–14.3 kPa, and only 48.1% in LSM >14 kPa. These results support the major role of LSM as a potential surrogate endpoint; however, liver stiffness lacks the power needed to identify patients requiring LT.^25^

In summary, although there are helpful prognostic scores that can identify patients with PSC at higher risk for liver-related death and transplantation, these tools are less effective at predicting short-term (months) transplant need compared with MELD and its derivatives. Given the heterogeneity of disease progression, integrating both short- and long-term risk scores may better identify patients at risk of clinical deterioration and those most likely to benefit from LT.

### Symptoms

Though not incorporated into current prognostic models, patient symptoms play a crucial role when assessing the need for LT. Patients with symptoms at diagnosis have a median survival to LT or mortality of 9 years, compared to 12 to 22 years in all patients with PSC^26^.^27^ Those with complications, including fatigue, pruritus, recurrent cholangitis, and an increased risk of malignancy, are not well captured by existing scores. However, these issues are of significant importance to patients and impact their quality of life.^28^ While revised Mayo and UK-PSC scores include variceal bleeding, there are no validated prognostic scores that include symptom burden. This is an important area for future study. Transplantation should be considered in patients with repeated life-threatening episodes of cholangitis and in those who have failed medical therapies for pruritus regardless of their markers of synthetic liver function and portal hypertension.

## Transplant indications

The main indications for LT in PSC are: 1) decompensated cirrhosis, and 2) cholangiopathic complications including bacterial cholangitis and refractory pruritus (Fig. 1). Jaundice can occur both in the setting of hepatic decompensation, as well as due to high-grade strictures or episodes of cholangitis, and indicates a worse prognosis.^14^ In some transplant centres, with strict selection criteria, CCA and even high-grade biliary dysplasia are indications. In a recent national survey from Italy, 51% of LTs were for hepatic decompensation, whereas 45% were for recurrent cholangitis.^9^ Decompensated cirrhosis – including ascites, hepatic encephalopathy, or variceal haemorrhage – remains the most common and widely accepted indication for LT in PSC. At the onset of a decompensating event, particularly ascites or hepatic encephalopathy, patients with PSC should be considered for LT referral. Following an index variceal haemorrhage, patients should be referred for LT if additional concerning features are present, such as other manifestations of hepatic decompensation or synthetic dysfunction that does not improve after initial management. Alternatively, it may be possible to closely monitor their trajectory before deciding on the requirement for LT.

### Cholangitis

Nearly 40% of those with PSC experience cholangitis.^14^ Recurrent bacterial cholangitis, often resulting from complex biliary strictures, is a driver of transplant referral, particularly when episodes are frequent or refractory to endoscopic and antimicrobial management.^29^ According to the United Network for Organ Sharing, patients with PSC and cirrhosis who have ≥2 hospital admissions within 1 year for acute cholangitis with documented bloodstream infection or evidence of sepsis (including haemodynamic instability requiring vasopressors) are eligible for MELD exception points. Bile duct bacterial isolates have been shown to be present in up to 58% of those transplanted for PSC and are associated with a shorter interval to LT.^30^ Cholangitis is not associated with increased waitlist mortality among patients with PSC.^31^ At our centres, patients are referred for LT consideration after two episodes of cholangitis within 1 year requiring inpatient admission despite endoscopic intervention, even in the absence of documented bloodstream infection.

### Pruritus

In a recent survey, 40% of those with PSC report pruritus that impairs quality of life.^32^ The PSC Support website reports similar estimates, with 36% of patients experiencing itch in the week preceding the survey.^28^ In those with worsening pruritus, imaging should be considered to assess for relevant strictures that could be treated endoscopically. Ursodeoxycholic acid (UDCA), the use of which is controversial in PSC, is ineffective for pruritus. Most recent EASL guidelines recommend bezafibrate as first-line treatment for pruritus in PSC, followed by rifampin and naltrexone.^27^ Further details on the safety and use of these treatments are provided in the waiting list section. In those in whom medical treatment is contraindicated or not effective,^14^ LT may be indicated. Clinical trials are currently exploring the role of ileal bile acid transport inhibitors for pruritus in PSC,^14^ with an open-label pilot study of marlixabat (a selective ileal bile acid transport inhibitor) demonstrating improvement in pruritus in PSC.^33^ Nasobiliary drains, which interrupt the enterohepatic circulation of bile, and plasmapheresis or molecular adsorbent recirculating system may be used as temporizing methods and can lead to symptomatic improvements.^34^^,^^35^ Though the prevalence of refractory pruritus as an indication for LT is not well documented, it is an accepted variant indication in many centres. Transplant referral for refractory pruritus should be considered when all available oral therapies have been exhausted.

### Malignancy

Individuals with PSC have an increased risk of malignancies, particularly CCA and colorectal cancer.^36^ The cumulative lifetime risk of CCA in PSC is estimated between 6% and 20%, with a pooled annual incidence of approximately 9.3 per 1,000 person-years.^37^ Notably, the highest risk is within the first year of PSC diagnosis at 2% with an annual incidence rate thereafter of 1.5%, highlighting the importance of early and ongoing surveillance.^3^^,^^38^^,^^39^ The risk of hepatocellular carcinoma (HCC) and gallbladder cancer is lower, with pooled incidence rates of 1.7 and 1.1 per 1,000 person-years, respectively.^37^

Diagnosing CCA in PSC is challenging due to non-specific clinical signs, overlapping imaging features, and limitations in diagnostic sensitivity. With the widespread use of LT for decompensated cirrhosis in PSC, CCA is a leading cause of death in PSC.^40^ In cohorts with low rates of CCA, performing surveillance MRI/MRCP may not detect CCA at an early enough stage to impact on survival.^41^ Endoscopic retrograde cholangiography (ERC) with brush cytology, FISH (fluorescence *in situ* hybridisation), and cholangioscopy with targeted biopsies, as described below, may be used selectively but lack definitive sensitivity for early detection.

Historically, the presence of CCA was considered an absolute contraindication to LT due to poor survival and high recurrence rates. However, the development of standardised neoadjuvant protocols, such as the Mayo Clinic protocol, have changed this paradigm.^42^ This approach is limited to patients with early perihilar CCA ≤3 cm without metastatic or nodal disease and involves external beam radiation, brachytherapy, chemotherapy, and staging laparoscopy followed by LT.^43^ Centres employing this strategy have reported 5-year post-transplant survival exceeding 60%.^44^ Consequently, European and American guidelines now support LT as a potentially curative option in centres experienced with transplant oncology and in highly selected patients with unresectable early stage perihilar CCA.^29^^,^^45^ These recommendations emphasise that LT should only be pursued in centres with experience in neoadjuvant therapy and transplant oncology.

In contrast, intrahepatic CCA remains a contraindication in most settings, though emerging data suggest that intrahepatic CCA (<2 cm) may be considered, ideally as part of a clinical trial and in conjunction with bridging therapies, such as ablation, transarterial chemoembolisation, or transarterial radioembolisation/selective internal radiation therapy.^29^^,^^45^^,^^46^

Controversies remain around the use of LT for patients with PSC and indeterminate strictures or high-grade dysplasia, which are suspected but not confirmed to harbour early malignancy. While some transplant programmes with short waiting lists consider patients with high-grade biliary dysplasia under MELD exception pathways, guidelines do not uniformly endorse this strategy.^29^

## Hepatobiliary evaluation during transplant assessment

### Blood markers

Routine transplant assessment in those with PSC should include assessment of liver function, liver enzymes, and tumor markers. At the point of LT assessment, the diagnosis of PSC is typically not in question. Still, it is important to take a thorough medical history to determine need for further investigations for secondary sclerosing cholangitis, particularly in patients without concomitant IBD and those with the small duct PSC subtype. This should include ischaemic, infectious, immune-mediated, chronic obstructive, toxic, and hereditary causes.^27^^,^^47^ IgG4 should be checked at the time of diagnosis in those with suspected PSC. Serum IgG4 more than 4 times the upper limit of normal, or IgG4:IgG1 >0.24 is strongly suggestive of IgG4-associated PSC.^48^ If not already done, a complete chronic liver disease workup should be performed to rule out concomitant or overlap disease. Increased cancer antigen 19-9 (CA 19-9) levels can support a diagnosis of CCA, but a normal level does not exclude malignancy.^49^ Further, CA 19-9 levels may increase in cases of biliary obstruction or inflammation, and approximately 10% of individuals are CA 19-9 non-secretors.^14^ Those with elevated serum IgG levels above the upper limit of normal have reduced transplant-free survival compared with those who have normal IgG levels.^50^

### Radiology

**Fig. 2:**
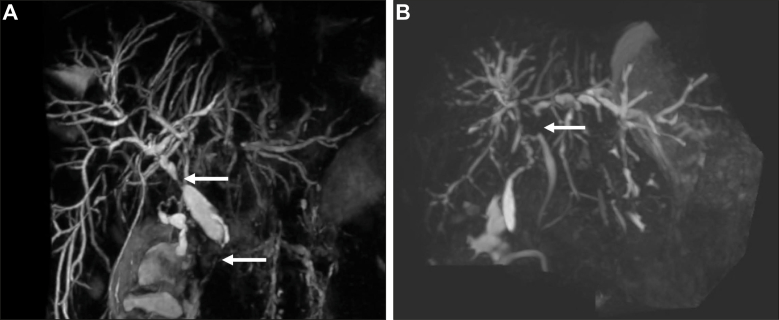
Magnetic resonance cholangiopancreatography in a patient with primary sclerosing cholangitis being considered for liver transplantation. (A) Arrows demonstrating high-grade strictures of common hepatic duct and lower common bile duct; (B) Arrow demonstrating high-grade stricture of common hepatic duct with left-sided intrahepatic duct dilatation. Biopsies demonstrated cholangiocarcinoma.

Indications for LT in patients with PSC are varied, as explored above, and the criteria for LT differ across the world. The diagnosis of PSC is usually not in question by the time the patient is being evaluated for LT. Therefore, the pre-transplant imaging work-up is aimed at evaluating the anatomical suitability for LT and the presence of any complications of PSC that could potentially affect or contraindicate LT (Fig. 2).

There are no defined criteria for what a radiological pre-transplantation work-up should entail. The EASL guidelines on PSC suggest an abdominal CT scan or MRI/MRCP scans within the last 6-12 months.^27^ Any other imaging work-up is driven by signs and symptoms (*e.g*. if the patient becomes jaundiced, an MRCP and contrast-enhanced MRI of the liver may be performed to look for high-grade strictures or CCA; if splanchnic thrombosis is suspected then contrast-enhanced imaging every 3 months may be appropriate).

If malignancy is not suspected and the patient has been listed due to decompensated cirrhosis, then the imaging evaluation with CT largely focuses on vascular anatomy and assesses for signs of a clinically occult malignancy. Conventional hepatic arterial anatomy is found in 55-76% patients, depending on the series,^51^ and while variant anatomy is not normally problematic for the transplant surgeon, it is important that any variants are highlighted. The CT will also assess patency of the portal and hepatic veins, as well as highlighting any large portosystemic shunts that may need ligating at the time of LT. MRCP can be used by the surgical team to plan the biliary anastomosis.

Given the prevalence of CCA in patients with PSC and the fact that elevated bilirubin may have prompted consideration for transplantation, detection of a CCA at the time of transplant work-up can occur. In patients with PSC, perihilar CCA is the most common type;^52^ however, distal (below the cystic duct) and intrahepatic CCAs also occur. The finding of an enhancing perihilar mass or focal periductal soft tissue should prompt urgent further evaluation for perihilar CCA with MRI/MRCP and ERC for cytological and/or histological analysis. The CT can accurately stage vascular involvement and evaluate for metastatic disease, while the MRI/MRCP can evaluate the length and radial diameter of the tumour. These factors help determine resectability and may inform LT suitability in some countries.

An enhancing intrahepatic lesion, unless demonstrating long-term stability or benign imaging features (*e.g*. of a haemangioma), should also prompt urgent further evaluation for an intrahepatic CCA, or possibly HCC.

### Upper GI endoscopy

Clinically significant portal hypertension may be present in up to 30% of those with PSC.^27^^,^^53^ Patients with LSM by VCTE <20 kPa and platelet count >150 × 10^9^/L are at very low risk (<5%) of having varices needing treatment and can safely avoid screening endoscopy.^54^ Though with small sample size, this rule has been validated in PSC, with a 0% false-negative rate for varices needing treatment using these criteria.^55^ Moreover, the Baveno VII guidelines affirm that non-selective beta blockers, especially carvedilol, can be initiated for the primary prevention of decompensation in patients with clinically significant portal hypertension, even in the absence of endoscopic confirmation.^54^ However, evidence is limited in PSC. When non-invasive criteria are met, carvedilol may be prescribed without the need for endoscopy.

### Hepatobiliary endoscopy

**Fig. 3:**
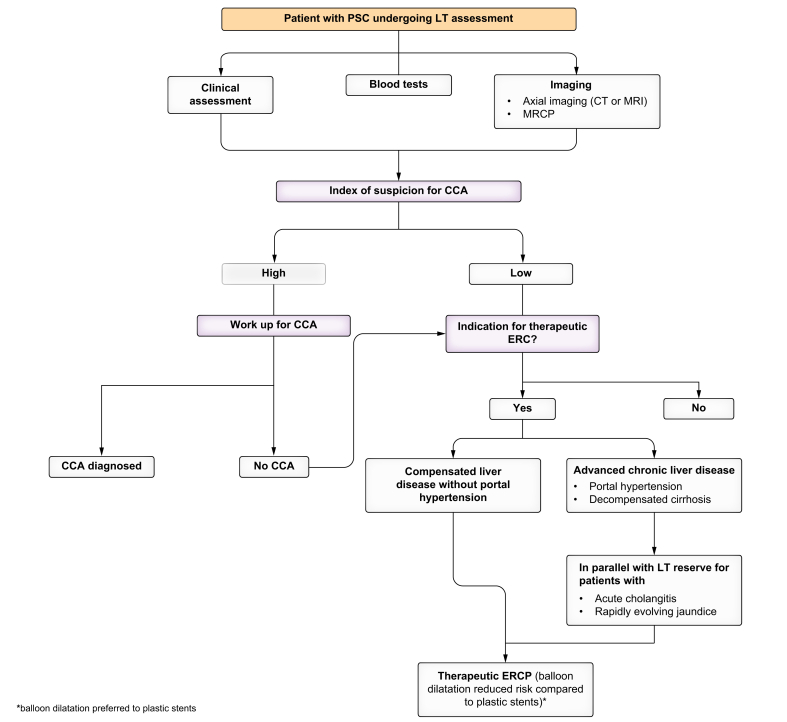
Endoscopic retrograde cholangiography in LT assessment. CCA, cholangiocarcinoma; ERC, endoscopic retrograde cholangiography; LT, liver transplantation; MRCP, magnetic resonance cholangiopancreatography; PSC, primary sclerosing cholangitis.

Although ERC is generally considered a therapeutic not a diagnostic procedure, it remains a key component in the evaluation of patients with PSC and suspected CCA (Fig. 3). Common indications for ERC in PSC include removal of choledocholithiasis or debris within the biliary tree, interrogation and evaluation of a high-grade biliary stricture, stent insertion for a malignant stricture and rarely insertion of a nasobiliary drain for the treatment of pruritus.^56^^,^^57^ Any decision to proceed with ERC in a patient with PSC should be made after careful multidisciplinary evaluation, as there is a significant risk of peri-procedural complications.^56^ Whilst there may not be an increased risk of the complications of pancreatitis, bleeding or perforation, the risk of cholangitis is threefold higher than in those without PSC.^58^ All patients should therefore receive peri- and post-procedural antibiotic prophylaxis for at least 1-3 days.^59^ Targets for intervention should be clearly defined before the procedure is undertaken, and the procedure should be undertaken by an experienced endoscopist.

In the peri-transplant assessment phase, evaluation of a high-grade stricture is a common clinical scenario. A triple A (Access, Assessment and Action) approach is recommended.^60^ Access involves selecting the appropriate accessories and guidewires. Assessment should be multi-modality, utilising biliary brush cytology and biopsy. Brush cytology is commonly performed at ERC and has an excellent specificity; however, it has a limited sensitivity of 43% in patients with PSC.^61^ In patients with a high index of suspicion of CCA and negative brushings, repeat ERCP and brushings, including incorporation of novel technologies, may be required to exclude CCA with more confidence. Adjuncts to brushing, such as FISH and next-generation sequencing, have shown promise in improving the sensitivity of these samples,^57^ although they are not in widespread clinical use. Single-operator cholangioscopy is widely available and allows for visualisation of the mucosa and targeted biopsies, with potential for application of AI technology.[62], [63], [64] However, it remains unclear whether it significantly increases the diagnostic yield of biliary dysplasia and cancer, and there is an increased risk of peri-procedural cholangitis.

A high-grade biliary stricture is defined as a >75% reduction of duct diameter in the common bile duct or hepatic ducts and warrants diagnostic work-up; whereas a relevant stricture is a high-grade biliary stricture with signs or symptoms of obstructive cholestasis and/or bacterial cholangitis. For relevant biliary strictures, first-line treatment is balloon dilatation, which has similar efficacy to short-term plastic stents but is associated with a lower complication rate.^65^ Patients with a higher Mayo risk score or a platelet count <150 × 10^9^/L are less likely to resolve jaundice after endoscopic intervention.^66^ An important observation of the DILSTENT study was that patients with more advanced disease were excluded, including those with a Child-Pugh score ≥8 and a Mayo risk score >2. Its applicability in patients undergoing transplant assessment is, therefore, unclear. In patients with more advanced liver disease, including portal hypertension, the safety and efficacy of therapeutic ERCP for relevant strictures should be considered, and it should be reserved for those with acute cholangitis or rapidly evolving jaundice, in parallel to a LT assessment.

The use of endoscopic ultrasound in the assessment of biliary strictures is an expanding field. Concerns regarding tumour seeding in potential transplant candidates appear to be historic with the advent of newer techniques (fine needle biopsy) and minimization of the number of passes.^67^ A key role of endoscopic ultrasound is in the evaluation of regional lymph nodes.

## Inflammatory bowel disease evaluation during transplant assessment

### Epidemiology and clinical features

IBD is present in approximately 60–80% of patients with PSC.^3^^,^^68^ The unique phenotype of PSC-associated IBD (PSC-IBD) is marked by extensive, often right-sided colitis, rectal sparing, backwash ileitis, and relatively mild symptoms despite extensive histological inflammation.^68^ The coexistence of these diseases impacts screening, immunosuppressive management, transplant planning, and post-LT outcomes. Despite the strong association between the two diseases, the relationship is incompletely understood, though it is thought to involve shared immunologic pathways and microbiome alterations.

### Colorectal cancer risk and surveillance

Due to the increased colorectal cancer risk, surveillance colonoscopy is necessary upon diagnosis of PSC, even in those who are asymptomatic.^68^ Current guidelines recommend annual colonoscopy with chromoendoscopy or segmental biopsies starting at the time of PSC diagnosis, irrespective of IBD duration or activity.^69^ In those with PSC without IBD, colonoscopy is recommended every 5 years, or sooner if symptoms arise.^56^ It is important to ensure that patients are up to date with the surveillance colonoscopy at the time of LT assessment. In a large, multi-institution retrospective cohort study of PSC-IBD, LT was associated with lower odds of colon dysplasia or cancer (odds ratio 0.66; 95% CI, 0.47–0.93).^70^ This association held even after adjustment for active disease, number of surveillance exams, and chromoendoscopy.

### Medical management of PSC-IBD

Management of stable IBD in PSC follows general IBD treatment principles. Immunomodulators (*e.g*. azathioprine, 6-mercaptopurine) are used both in moderate disease and in post-LT immunosuppressive regimens. Observational data suggest these agents may be associated with a reduced risk of acute cholangitis and longer IBD remission after LT.^71^ Conversely, anti-TNF therapy has been associated with an increased risk of acute cholangitis in PSC-IBD,^71^ raising concerns about its use in those with recurrent cholangitis as their transplant indication. Vedolizumab, a gut-selective anti-integrin, appears safer, with studies demonstrating clinical improvement in over half of treated patients, although it does not appear to modify the liver disease course or significantly improve liver biochemistry.^72^ Patients with PSC-IBD were not included in the seminal trials with the newer advanced therapies (IL-12/23, S1P, and JAK inhibitors), and the safety and efficacy of these therapies in PSC-IBD will be guided by emerging real-world data.^73^

### Surgical considerations and timing of colectomy

For patients with active or refractory IBD, especially those approaching LT, optimizing disease control is essential in keeping with IBD society guidelines. The decision to continue medical management *vs*. proceed to surgery should involve multidisciplinary input from gastroenterology, hepatology, and transplant teams.^74^ Active IBD at the time of LT is associated with worse graft outcomes and an increased risk of rPSC.^75^ A multicentre study found an increased hazard of rPSC in those with active disease post-LT (HR 1.7; 95% CI 1.08-2.75).^76^

**Fig. 4:**
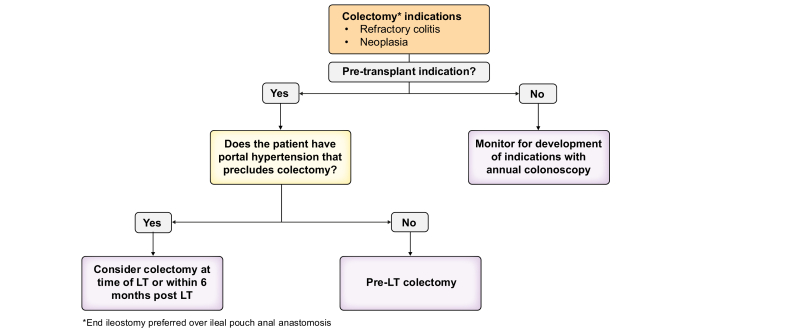
Flowchart for colectomy timing in PSC-IBD during LT work up and assessment. ∗End ileostomy preferred over ileal pouch anal anastomosis. PSC-IBD, primary sclerosing cholangitis-inflammatory bowel disease; LT, liver transplantation.

The timing of surgery relative to LT should consider the urgency of transplant, severity of colitis, and presence of dysplasia or colorectal malignancy. The ESOT (European Society of Organ Transplantation) recommends a multidisciplinary approach and supports pre-transplant colectomy in select patients with refractory colitis or neoplasia.^74^ Patients with persistently active disease despite escalation may require colectomy prior to or at the time of transplantation, particularly if there is no contraindication from a portal hypertension perspective. Historically, the risk of rPSC was shown to be significantly less in those who undergo pre/peri-transplant colectomy (3.4%) than in those who undergo post-LT colectomy (43.8%),^77^ with meta-analysis demonstrating a lower hazard of rPSC in those who undergo pre-transplant colectomy (HR 0.65; 95% CI 0.42-0.99).^78^ However, a more recent international multicentre study did not find a significant difference in rPSC based on timing of colectomy in PSC-IBD (HR 1.57; 95% CI 0.54-4.6).^79^ The indication for colectomy influences the risk of rPSC. Medically, patients with refractory PSC-IBD have a higher incidence of rPSC and biliary complications than those undergoing colectomy for oncological indications.^80^ In those in whom colectomy is contraindicated due to clinically significant portal hypertension pre-LT, colectomy should be considered within 6 months post-LT (Fig. 4).^81^

### Choice of surgical technique

Ileal pouch anal anastomosis (IPAA) should be used cautiously in PSC-IBD due to an increased risk of pouchitis, particularly after LT, where chronic pouchitis has been reported in over 67% of cases.^82^ Colectomy with end ileostomy has consistently been associated with more favourable graft outcomes compared with IPAA;^83^ however, a recent multicentre study reported comparable graft survival between IPAA and end ileostomy.^80^ Ultimately, the surgical approach should be determined by a multidisciplinary team – including surgeons, hepatologists, and gastroenterologists – and take patient preferences into account, although we generally recommend avoiding IPAA in PSC-IBD.

## Listing considerations

### Graft options

Donation after brain death (DBD) grafts are preferred in PSC due to superior graft survival and lower rates of ischaemic cholangiopathy. Multiple studies have shown that donation after circulatory death (DCD) grafts are associated with increased risks of graft failure and biliary complications, particularly ischaemic cholangiopathy.[84], [85], [86] However, DCD may be reasonable in selected patients – such as those who are older, have a low MELD score, and are at low risk of prolonged ischaemia – when high-quality DBD grafts are unavailable.^84^^,^^86^^,^^87^ Optimal DCD outcomes depend on minimising donor warm ischaemia time and carefully selecting both donor and recipient. For patients with PSC, particularly younger or high-risk individuals, caution remains warranted when considering DCD grafts.^87^ Because MELD and its derivatives may underrepresent disease severity and disadvantage patients with PSC, living donor liver transplantation (LDLT) is an important option for eligible patients with an available donor.^88^ This can shorten waiting list times and decrease waiting list mortality.^89^^,^^90^ A large registry study identified reduced graft survival with LDLT compared to DCD and DBD.^91^ In a large multicentre study, post-LT survival was inferior with LDLT compared to DBD (HR 1.92; 95% CI 1.05-3.49),^92^ making DBD preferable if available.

Special considerations need to be evaluated in concomitant PSC-IBD, as management can be challenging. In patients who have undergone bowel surgery before LT, adhesions, together with severe portal hypertension, may increase surgical risk. That said, a combined procedure of LT and total colectomy seems to be a viable option for patients with PSC-IBD.

There is no definitive consensus on the ideal biliary reconstruction technique in LT for patients with PSC. Historically, many centres have preferred hepaticojejunostomy over duct-to-duct anastomosis due to the perceived lower risk of disease recurrence, biliary complications and *de novo* CCA. Duct-to-duct anastomosis restores the natural anatomy and function of the biliary tree, while maintaining normal Oddi sphincter function, and facilitates ERC access, which can be useful in the post-transplant period. Additionally, in patients with previous bowel surgery resulting in a shortened small bowel or small bowel oedema due to portal hypertension, hepaticojejunostomy may not be feasible.^93^^,^^94^ However, it is not clear whether hepaticojejunostomy is superior to duct-to-duct anastomosis with respect to survival benefit. A meta-analysis showed that anastomotic bile leak rates, graft survival, PSC recurrence and *de novo* CCA were comparable between the two reconstruction techniques.^95^ However, hepaticojejunostomy seems to be associated with higher risks of acute cholangitis^96^*.* The recent ESOT Consensus Statement*,* although with moderate quality of evidence, recommended that the choice of biliary anastomosis should be left to the surgeon; however, duct-to-duct anastomosis is recommended as the reconstructive technique of choice whenever technically feasible.^74^

Novel preservation technologies may abrogate the increased risks of post-transplant complications in patients with PSC. *Ex situ* machine perfusion, namely hypothermic oxygenated perfusion and normothermic machine perfusion, have both demonstrated the capacity to optimise graft assessment and selection, facilitate transplant logistics, and achieve improved clinical outcomes.^97^^,^^98^ While machine perfusion may improve outcomes in patients with PSC, given its promising early outcomes, long-term evaluations of these techniques are still needed.^98^

### Exception points

Allocation of exception points varies by transplant centre and governing body. As MELD and its derivatives do not fully capture disease burden in variant transplant indications, exception points are awarded in some geographic areas to address clinical scenarios underrepresented by MELD.

In the UK there is a national transplant waiting list, and the National Liver Offering Scheme was introduced in 2018 for elective DBD transplantation.^99^ This allocates organs according to the transplant benefit score, which incorporates recipient and donor characteristics, including aetiology of liver disease. For refractory pruritus and recurrent cholangitis, and for patients being considered under a new service evaluation of transplantation for an unresectable intrahepatic cholangiocarcinoma (≤2 cm) without extrahepatic disease and without underlying chronic liver disease, patients can be listed as having a variant syndrome and offered organs outside of the transplant benefit score according to waiting time.^100^

In Canada, exception point allocation remains highly variable across provinces. A recent survey revealed that four of five provinces grant exception points for recurrent cholangitis, typically based on frequency of hospitalisations and antibiotic use.^101^ Ontario is the only province with specific criteria for awarding points for refractory pruritus, while three provinces recognise CCA as an indication, with differing criteria and exception points awarded.^101^ The absence of national consensus creates regional disparities.

In the US, the Organ Procurement and Transplantation Network has developed detailed guidance for MELD exception review. For recurrent bacterial cholangitis, exception points may be approved when there are ≥2 documented hospitalisations within 6 months despite maximal medical therapy.^102^ For refractory pruritus, MELD exceptions can be considered when it significantly impairs quality of life and is unresponsive to medical therapy.^102^ Evidence of complications such as skin excoriations or sleep deprivation is often required. For early-stage perihilar CCA, standardised protocols (*e.g*. the Mayo protocol) allow MELD exception listings, typically granting patients a starting MELD score of 22, with upgrades every 3 months.

## Waiting list management

### Surveillance bloods and imaging

In our centres, surveillance blood tests are performed monthly to monitor for infection, changes in liver function or enzymes, and kidney function. The tumour marker alpha-fetoprotein is measured every 6 months as part of HCC surveillance in patients with cirrhosis. Outside of the transplant waiting list setting, imaging surveillance of PSC is generally recommended by international guidelines,^27^^,^^45^ but is somewhat controversial. A recent prospective study of combined clinical evaluation, tumour markers, and imaging evaluation failed to diagnose CCA early enough to improve survival.^41^ Imaging surveillance on the transplant waiting list is a distinct scenario and is also not guided by high-quality evidence.

Imaging surveillance is guided by the indication for LT and symptom development. For example, patients who have received neoadjuvant chemoradiotherapy prior to LT for CCA require close surveillance with 3-monthly contrast-enhanced cross-sectional imaging. Other factors to consider are local resources as well as the expected time on the LT waiting list. In the case of portal vein thrombosis identified on pretransplant assessment, active surveillance and management are critical. Cross-sectional imaging should be repeated every 3 months to monitor for clot regression, stability, or progression, and treatment should be guided by imaging findings (*i.e*. continuation of anticoagulation alone *vs*. consideration of portal vein recanalisation with transjugular intrahepatic portosystemic shunt placement).^103^ Overall, imaging surveillance is guided by the indication and symptom development, as is the case for patients with PSC who are not on the LT waiting list.

### Mental health

Mental health is an essential part of patient management while on the LT waiting list. In a 2020 survey through PSC Support, 75% of respondents indicated that psychological support was very or extremely important on the waiting list, with 69% indicating they needed psychological support on the waiting list.^104^ Further, 54% experienced anxiety. A 2007 study identified that those on the LT waiting list had more depression and lower health-related quality of life compared to healthy controls.^105^ The 2007 study did not find a significant difference in mental health in those with chronic liver disease on or off the LT waiting list,^105^ supporting that mental health should be addressed while on the waiting list and as part of every clinic appointment, with psychological and psychiatric support offered. Patients can be directed to local accredited PSC support and information websites for reliable information.

### Nutrition and prehabilitation

Physical frailty/sarcopenia contributes to adverse clinical outcomes before and after LT.^106^ The liver frailty index was developed for use in patients with cirrhosis in the ambulatory setting and is associated with waiting list mortality.^107^ Prehabilitation has been demonstrated to improve post-surgical outcomes and should be offered to patients.^108^^,^^109^ Nutritional support is an integral part of LT care.^110^ Those with PSC, and particularly those with PSC with advanced chronic liver disease, are at increased risk of nutritional deficiencies, including fat soluble vitamin deficiencies (vitamins D, A, K, and E), calorie deficits, and macronutrient deficiencies (protein, fat, and carbohydrates).^111^^,^^112^ Oral nutritional supplementation may be required to meet the recommended intake of 35-40 kcal/kg/day and 1.2-1.5 g/kg/day of protein and provide macronutrients. If these measures are insufficient, dietician involvement and enteral feeding should be considered.^112^

### Rotating antibiotics

Rarely, those with recurrent bacterial cholangitis may require long-term and rotating antibiotics.^113^ There is no strong evidence to support the use of rotating antibiotics in recurrent cholangitis. Use may increase the risk of multidrug resistance. Still, society guidelines acknowledge the role in select cases with expert multidisciplinary input to drive decision making and choice of antibiotic, considering sensitivity from microbiological samples, including biliary aspirates.^56^

### Pruritus management

Pruritus is associated with markedly decreased quality of life, including impaired social and physical function and adverse mental health. Consequently, pruritus must continue to be managed while on the transplant waiting list. If pruritus worsens, relevant strictures should be investigated with cholangiography and balloon dilatation with brushings.^114^ Non-pharmacologic approaches begin with keeping nails short, using emollients, and avoiding hot showers and baths.^27^ First-line pharmacotherapy is bezafibrate, with rifampin, naltrexone, and sertraline used as second-, third-, and fourth-line therapies, respectively.

Bezafibrate, a PPAR (peroxisome proliferator-activated receptor) agonist, has been demonstrated to reduce itch by ≥50% in 45% of patients compared to 11% with placebo over 3 weeks, with significant improvements in quality of life metrics.^115^ Individuals with Child-Pugh A and B were included in the trial. Safety data for fibrates in cirrhosis are scarce.^116^ Though liver disease is listed as a contraindication in the product monograph, we regularly prescribe bezafibrate for cholestatic pruritus in Child-Pugh A and B cirrhosis. We recommend use with caution in Child-Pugh C cirrhosis, particularly in those with progressive renal dysfunction. Bezafibrate should not be used when creatinine clearance falls <60 ml/min, though some decrease the dose to 200 mg with frequency guided by extent of renal dysfunction. We monitor creatinine kinase 1 month after initiation and repeat if symptoms of myopathy develop, including muscle weakness or pain.

Rifampin, an activator of the pregnane X receptor (PXR), is effective but requires monitoring for hepatotoxicity. In a large retrospective cohort study of rifampin-associated hepatotoxicity in patients treated for cholestatic pruritus, fewer than 5% developed hepatotoxicity.^117^ Of the five who did, two had index bilirubin >2.5 mg/dl. AASLD primary biliary cholangitis guidance suggests avoiding rifampin in those with a bilirubin >2.5 mg/dl.^118^^,^^119^ In some who do prescribe above this threshold, co-prescription of vitamin K can be given to reduce the risk of coagulopathy.^120^ With weekly monitoring of liver tests due to variable drug metabolism, we prescribe rifampin in decompensated cirrhosis. In those without cirrhosis and those with compensated cirrhosis, we monitor liver enzymes every 1-4 weeks for the first 3 months after initiation and discontinue if alanine aminotransferase rises to more than 5 times the upper limit of normal. The half-life of rifampin is approximately 2-3 h. As rifampin is a strong CYP 3A4 inducer, medication interactions should be reviewed prior to initiation. Immediate post-transplant calcineurin inhibitor levels may be increased due to rifampin use.

Opioid antagonists (*e.g*., naltrexone) and the selective serotonin reuptake inhibitor sertraline may be helpful, but the evidence is modest and mostly derived from small or uncontrolled trials.^121^^,^^122^ Historically naltrexone was avoided in those with cirrhosis due to concerns regarding hepatotoxicity. However, in a large retrospective cohort study of those with compensated and decompensated cirrhosis and alcohol use disorder, naltrexone was shown to be safe.^123^ The half-life of naltrexone is 4-13 h. Dosing should start at 12.5 mg daily with up-titration every 3-7 days to a maximum of 50 mg per day.[124], [125], [126] Naltrexone should not be given in those with concurrent opioid use. Further, as opioids are likely to be used as part of a post LT pain management strategy, alternative anti-pruritic medications should be considered. For patients already stabilised on naltrexone, anaesthesiology should be engaged at listing to plan opioid alternatives for intra- and postoperative pain management.

Sertraline accumulates in the setting of hepatic impairment. In those with Child-Pugh A or B cirrhosis, sertraline should start at a low dose of 25 mg with slow titration up by 25 mg each time and no sooner than every 2 weeks to a maximum dose of 100 mg.^127^ Sertraline is advised against in Child-Pugh C cirrhosis. Sertraline may increase bleeding risk due to selective serotonin reuptake inhibitor-induced platelet serotonin depletion.^128^

Bile acid sequestrants are occasionally used (*e.g*. cholestyramine), though there is insufficient evidence in PSC, and their tolerability and palatability are limited.

### Ursodeoxycholic acid

UDCA increases the hydrophilic pool of bile acids, stabilises cell membranes, inhibits apoptosis, and improves bile flow.^129^ If a patient is stable and tolerating low-intermediate dosing, there is insufficient evidence to support either continuation or discontinuation during LT assessment and listing.

In general, there is robust evidence to support the role of UDCA in improving liver biochemistry.[130], [131], [132], [133] However, meta-analyses have not demonstrated improvement in survival, prevention of CCA, disease progression, nor symptom improvement with UDCA.[131], [132], [133], [134] A large Japanese retrospective cohort study of 325 patients with PSC identified improvement in transplant-free survival with low-dose UDCA. However, the UDCA monotherapy group was younger (mean age 45.8 compared to 52.5), had less occurrence of symptoms (34% compared to 40%), and had lower FIB-4 (1.14 compared to 1.98) than the untreated group at baseline.^135^ These estimates were not directly statistically compared as the Japanese study included a bezafibrate group and a combination bezafibrate/UDCA group, and all comparisons were omnibus tests. EASL guidance indicates UDCA can be used at 15-20 mg/kg/day with available data not allowing for a firmer recommendation. AASLD guidelines note UDCA can be considered after 6 months of observation prior to assessing if ALP and gamma-glutamyltransferase spontaneously normalise. Higher doses (28-30 mg/kg/day) should be avoided due to their association with worse outcomes including development of varices, need for LT, and death.^136^ Further, higher doses are associated with colonic neoplasia in those with PSC-IBD.^137^ Still, the pathogenic mechanisms of bile acids in the colon remain unclear and these adverse associations have not been observed at low or medium doses. In our centres, UDCA is not initiated after listing; however, if a patient is already taking UDCA without side effects, it is continued.

## Post-transplant management

### Risk of disease recurrence

In a recent systematic review and meta-analysis of 14 studies, the pooled prevalence of rPSC was 17.7%.^78^ A large French national cohort study of 571 patients from 1985-2019 reported an rPSC rate of 25.9%, with 5-, 15-, and 25-year recurrence risks of 15.6%, 37.9%, and 52.6%, respectively.^138^ The diagnosis is based on histologic (fibrous cholangitis and/or fibro-obliterative lesions with or without ductopenia, biliary fibrosis, or biliary cirrhosis) or imaging findings typical of cholangiopathy 90 days or more post-LT, after excluding alternative causes of biliary strictures (including hepatic artery ischaemia, DCD cholangiopathy, ABO incompatibility, ductopenic rejection, and cytomegalovirus).^139^^,^^140^ The presence of refractory IBD, CCA pre-LT, recurrent cholangitis as the indication for LT, advanced donor age, acute cellular rejection, cytomegalovirus, and increased systemic inflammatory states, both before and after,^76^ have been identified as risk factors for rPSC.^141^^,^^142^ Periductal infiltration with IgG4 cells in the explant is associated with increased risk of rPSC and shorter time to re-transplant.^143^ Recently, a study including Canadian and UK cohorts demonstrated that the development of cholestasis within 3-12 months following LT was predictive of rPSC and graft loss.^144^ There is no role for protocol biopsies, though abnormal liver biochemistry should prompt liver biopsy if clinically indicated.^140^ As rejection is associated with rPSC, rejection should be promptly acted upon with histologic confirmation and treatment initiation.^145^ Cross-sectional imaging should be performed to evaluate the hepatic artery and bile duct anatomy in the setting of cholestatic liver enzyme elevation.

As described above, the role of colectomy pre-LT is an ongoing topic of study, though it has been associated with rPSC. Active management of IBD should remain a priority post-LT to decrease the risk of rPSC. Though rare, *de novo* CCA can develop in those with rPSC.[146], [147], [148] Our local practice is to perform annual MRCP once rPSC has been diagnosed, to identify the rate of progression of cholangiopathy, targets for biliary intervention and areas suspicious for CCA.

There are no approved disease-modifying therapies to decrease the risk of rPSC. There is insufficient data to support the use of UDCA after LT, with a large French cohort demonstrating UDCA had no impact on rPSC risk.^138^ We do not start UDCA for prevention of rPSC. However, in those who develop rPSC with elevated ALP, a trial of 15-20 mg/kg/day can be given and continued if biochemical improvement is observed.^140^

PSC recurrence is associated with the need for repeat LT, with the European Liver Transplant Registry demonstrating increased odds of re-transplant in those with rPSC compared to those without rPSC (odds ratio 3.6; 95% CI 2.7-4.8).^79^ Further, this large registry identified worse graft and overall survival in those with rPSC, though this impact was altered by timing of recurrence, with better graft survival in those who were diagnosed with rPSC 5 years after LT (15-year graft survival of 38% in those diagnosed with rPSC within 5 years compared to 51% in those diagnosed after 5 years).^79^ Mortality at 90 days is similar for patients undergoing re-LT for rPSC compared with other indications; however, mortality at 5 years is significantly lower.^149^

### Post-transplant immunosuppression

There is no universally agreed immunosuppression strategy post-LT for PSC, and the choice should be individualised. Due to the increased risk of rPSC associated with T cell-mediated rejection,^140^ some centres favour an enhanced immunosuppression strategy; however, there is a great deal of variability across centres.^113^ Induction with basiliximab and/or steroids is recommended.^140^ The backbone of long-term immunosuppression post-LT for PSC is tacrolimus. Tacrolimus is the preferred calcineurin inhibitor, as it offers superior long-term patient and graft survival compared to cyclosporine.^150^ However, tacrolimus was associated with rPSC, independent of IBD activity and previous colectomy, in a large multicentre study.^151^ In this study, however, low dose steroids were part of the immunosuppression strategy. Other large studies have not identified a similar association.^76^^,^^138^ Use of steroids has been associated with rPSC^152^ in multivariate analysis; however, it is unclear whether this reflects active IBD or rejection, both of which are independent risk factors for rPSC. Univariate analysis in a large cohort study identified steroid use as an independent risk factor for rPSC (HR 2.22; 95% 1.49-3.31). Corticosteroids should be tapered early unless there are overlap features with autoimmune hepatitis.^138^^,^^153^ Adjunct therapies, including mycophenolate and azathioprine can be used and tapered after the first year.^140^ Mycophenolate is associated with a decreased hazard of rPSC (HR 0.62; 95% CI 0.41-0.92); however, after adjustment for covariates, this association was no longer statistically significant.^138^ In our centres, tacrolimus is the preferred first-line option for immunosuppression.

### Post-transplant IBD management

A systematic review and meta-analysis of 13 studies identified that in those with intact colons at the time of LT, IBD improved in 29.4%, remained unchanged in 51.4%, and worsened in 25.2% post-LT.^154^ The cumulative incidence of an exacerbated -post-LT IBD course was 0.22.^154^ Active IBD at the time of LT is associated with worse post-LT disease course.^155^ Management of IBD should be undertaken in collaboration with specialist IBD and transplant hepatology teams. Biologics and small molecules are effective for the treatment of IBD after LT.[156], [157], [158] However, biologic therapy in combination with standard post-LT immunosuppression is associated with increased infection risk,^159^ particularly with *Clostridioides difficile*.^160^ Further, in a multicentre study, 17% of patients on anti-TNF after LT developed colorectal cancer, supporting vigilant post-LT annual colonoscopies in those with PSC-IBD.^161^ In those without PSC-IBD at LT, there should be a low threshold to investigate for IBD after LT if symptoms develop, as *de novo* IBD can develop after LT (26.2% of patients without IBD at time of LT developed IBD over a median time of 11.7 years in a US-based cohort).^162^

Data on optimal post-LT immunosuppression in PSC-IBD are conflicting. The use of tacrolimus and mycophenolate have been independently associated with increased IBD activity after LT.[162], [163], [164] However, these findings are not consistent across studies.^165^ Azathioprine, and the combination of azathioprine and cyclosporin are associated with decreased risk of IBD activity after LT.^162^^,^^164^^,^^166^ In PSC-IBD, we prefer azathioprine over mycophenolate. Due to improvement in graft and overall survival, tacrolimus remains the mainstay of immunosuppression.

### Risk of post-transplant malignancy

Patients transplanted for PSC are at risk of *de novo* malignancies after transplant with a 10-year cumulative incidence of cancer of 18.7%.^167^ The most common malignancies after transplant for PSC are post-transplant lymphoproliferative disorder (PTLD), renal cell carcinoma, and colon cancer.^167^ The immunosuppressive regimens were not significantly different between patients with PSC and cancer and those without; however, the immunosuppression intensity have not been directly compared. PTLD risk is linked to greater intensity of immunosuppression, so a judicious balance of immunosuppression is a priority, acknowledging that T cell-mediated rejection is associated with rPSC. Clinical screening for signs and symptoms of PTLD should be performed at each clinical visit. Apart from standard age-based cancer screening and regular skin surveillance there is no clear guidance on who and how to screen for post-transplant malignancies.

In a systematic review and meta-analysis, colon cancer risk after LT for PSC was 5.8 per 1,000 person-years.^168^ This incidence markedly increased in those with PSC-IBD and an intact colon to 13.5 per 1,000 person-years.^168^ Dysplasia was more common in patients who developed rPSC after transplantation (54% of those with dysplasia had rPSC compared to 38% who did not develop dysplasia).^70^ Surveillance with colonoscopy should continue annually after transplant in patients transplanted with PSC-IBD.

## Conclusion

In conclusion, LT remains the only definitive treatment for PSC. Given the progressive nature of the disease and the risk of complications, prognostic scores can be valuable in identifying patients at increased risk of decompensation or adverse outcomes. These can help guide the decision to proceed with LT. The main indications for LT in PSC include decompensated cirrhosis, cholangiopathic complications (including recurrent cholangitis and refractory pruritus), and CCA in selected programmes. While MELD and its derivatives are widely used for assessing liver transplant eligibility, they do not always fully capture the severity of PSC, especially in patients with variant LT indications. In such cases, exception points should be considered to more accurately reflect the disease burden and provide a mechanism for successful LT.

As the landscape of PSC management evolves, further standardisation of transplant referral criteria and MELD exception pathways is critical to improve equity and access to LT. Multidisciplinary and personalised care involving the patient, hepatology, gastroenterology, radiology, and transplant surgery remain essential – particularly in the management of PSC-IBD. Future research should prioritise prospective studies aimed at personalising transplant referral and listing strategies, clarifying optimal PSC-IBD management, refining post-LT immunosuppressive strategies to mitigate recurrence, and exploring the role of PSC-specific prognostic scores in transplant listing.

## Abbreviations

ACLD, advanced chronic liver disease; ALP, alkaline phosphatase; AST, aspartate aminotransferase; CA 19-9, cancer antigen 19-9; CCA, cholangiocarcinoma; DCD, donation after circulatory death; DBD, donation after brain death; ERCP, endoscopic retrograde cholangiopancreatography; HCC, hepatocellular carcinoma; HOPE, hypothermic oxygenated perfusion; IBD, inflammatory bowel disease; IPAA, ileal pouch-anal anastomosis; LDLT, living donor liver transplantation; LSM, liver stiffness measurement; LT, liver transplantation; MELD, model for end-stage liver disease; MRCP, magnetic resonance cholangiopancreatography; NMP, normothermic machine perfusion; PSC, primary sclerosing cholangitis; PSC-IBD, primary sclerosing cholangitis-inflammatory bowel disease; rPSC, recurrent primary sclerosing cholangitis; UDCA, ursodeoxycholic acid; UKELD, United Kingdom model for end-stage liver disease; ULN, upper limit of normal; VCTE, vibration-controlled transient elastography.

## Authors’ contributions

ADF, JSN, AP, MS, MDS, AJML, and DJ conducted the literature review, and drafted the manuscript. DJ conceptualized the review. All authors provided critical revisions and approved the final version of the manuscript.

## Declaration of generative AI and AI-assisted technologies in the writing process

During the preparation of this work the author used ChatGPT to improve grammar and language clarity during manuscript preparation. After using this tool/service, the authors reviewed and edited the content as needed and take full responsibility for the content of the publication.

## Financial support

No financial support was received to produce this mansucript.

## Conflict of interest

Please refer to the accompanying ICMJE disclosure forms for further details.
